# Zebrafish as a Vertebrate Model System to Evaluate Effects of Environmental Toxicants on Cardiac Development and Function

**DOI:** 10.3390/ijms17122123

**Published:** 2016-12-16

**Authors:** Swapnalee Sarmah, James A. Marrs

**Affiliations:** Department of Biology, Indiana University-Purdue University Indianapolis, Indianapolis, IN 46202, USA

**Keywords:** zebrafish in cardiotoxicity research, environmental toxicity, cardiotoxicity, non-genetic causes of congenital heart defects, congenital heart defects, zebrafish

## Abstract

Environmental pollution is a serious problem of the modern world that possesses a major threat to public health. Exposure to environmental pollutants during embryonic development is particularly risky. Although many pollutants have been verified as potential toxicants, there are new chemicals in the environment that need assessment. Heart development is an extremely sensitive process, which can be affected by environmentally toxic molecule exposure during embryonic development. Congenital heart defects are the most common life-threatening global health problems, and the etiology is mostly unknown. The zebrafish has emerged as an invaluable model to examine substance toxicity on vertebrate development, particularly on cardiac development. The zebrafish offers numerous advantages for toxicology research not found in other model systems. Many laboratories have used the zebrafish to study the effects of widespread chemicals in the environment on heart development, including pesticides, nanoparticles, and various organic pollutants. Here, we review the uses of the zebrafish in examining effects of exposure to external molecules during embryonic development in causing cardiac defects, including chemicals ubiquitous in the environment and illicit drugs. Known or potential mechanisms of toxicity and how zebrafish research can be used to provide mechanistic understanding of cardiac defects are discussed.

## 1. Introduction

Cardiovascular disease is the leading cause of death globally [1]. According to the World Heart Federation’s report, a total of 17.3 million people die every year due to heart disease [2]. Congenital heart defects (CHDs), a group of cardiovascular disorders describing malformations of the heart’s structure existing at birth are the most common congenital defects worldwide. There are 25 known types of CHDs [3], but little is known about the etiology of these defects. Genetic factors, environmental factors, and a combination of both of these factors can produce CHDs. Heart development is particularly sensitive to environmental contaminant exposure. Environmental pollution is a major problem of the modern world and a major public health concern. Industrialization and urbanization have been expanding dramatically due to advances in science and technology, but at the same time, increased accumulation of toxic by-products in air, soil, and water pose serious threats to human health. Toxicants are often ingested knowingly as substances of abuse, which threaten the health of children during gestation. Environmental pollution affects the health of all age groups; however, exposure to pollutants during early development is particularly problematic.

Although there are many air and water pollutants that have been assayed and verified as potential agents for cardiac toxicity, including CHDs [4], there are many new chemicals that have become ubiquitous in the environment with little or no risk assessment information available. In recent decades, engineering of nanomaterials has increased considerably because of the unique properties and utilities of nanoparticles in medicine, optics, and electronics. However, with increased utilization and exposure to nanoparticles in the environment, humans have an increasing risk, which will require assessment of their potential impact on health. Multiple reports have highlighted the toxic effects of nanoparticles that lead to serious illness and developmental defects [5]. Abundant use of pesticides (i.e., herbicide, insecticide, and fungicide) on farms, fields, and at home, cause accumulation of these compounds in the environment. Although insecticides, fungicides, and herbicide benefit society and the economy because they effectively kill insects, fungi, or weeds, respectively, they are potent compounds that often interfere with essential biological processes. For example, widely used organophosphate and carbamate insecticides such as chlorpyrifos, malathion, diazinon, carbaryl, carbofuran, and methomyl are potent acetylcholinesterase inhibitors. Most herbicides are strong acids, amines, esters, or phenols. Because of their widespread use, these potent chemicals are ubiquitous in the environment, accumulating in soil and water bodies. Concentration of organic pollutants, such as polycyclic aromatic hydrocarbons and halogenated compounds, are consistently increasing in the environment due to increased industrial processes and incomplete combustion of carbon, widely used flame retardants, and naturally, from volcanos and forest fires. Dioxin, a class of heterocyclic 6-membered ring compounds, are persistent environmental pollutants. Because of their chemical stability, dioxins accumulate in the environment, enter the food chain, and concentrate in fat tissue. Likewise, halogenated carbazole derivatives, a class of compounds containing tricyclic aromatic heterocycles, are reportedly increasing in the environment, specifically in rivers, lake sediments and soils [6,7,8,9]. Polycyclic aromatic hydrocarbons like benzopyrene (a compound generated from incomplete combustion of carbon) and phenanthrene (the most abundant component of coal tar) are ubiquitous contaminants. Flame retardants, such as Firemaster 550 and polybrominated diphenyl ethers are also abundant in the environment. However, very little information is available on the toxicity upon chronic exposure to these compounds, specifically whether exposure during prenatal development can cause CHDs.

In addition to environmental pollutants, use of recreational drugs like cocaine, heroin, methamphetamine, uncontrolled alcohol consumption, and smoking are known to cause cardiovascular diseases including CHDs. Substance abuse is associated with cardiovascular diseases, including myocardial infarction, heart failure, stroke, cardiomyopathy, arrhythmias, and many other disorders. In utero exposure to these substances can lead to various defects in the fetus, including different forms of CHDs.

There is an urgent need for reliable model systems to study the effects of exposure to these substances in animals, to understand the mechanisms of the defects and to explore strategies to mitigate those defects. Zebrafish has long been used to study developmental biology and molecular genetics. Zebrafish studies have resulted in novel insights into the molecular regulation of vertebrate cardiac development and human cardiovascular diseases because mechanisms of cardiac development are highly conserved among vertebrates [10]. Researchers used zebrafish to study the effects of chemicals on cell division and differentiation in the 1950s [11,12]. There have been great efforts to establish zebrafish as a toxicology model organism [13,14,15,16]. Cardiovascular defects in response to various stressors and toxicants have been characterized using zebrafish, illustrating the potential utility of the zebrafish model [17,18]. However, the full utility of zebrafish as a toxicology model is still being realized. Zebrafish provides an outstanding opportunity to dissect cellular and molecular cardiac development mechanisms interrupted by exposure to various chemicals.

Here, we highlight the advantages of using zebrafish in cardiotoxicity research and review literature studying the effects of various environmental pollutants, as well as drugs of abuse, on cardiovascular development and function. This review highlights a variety of toxicants that have been studied, particularly studies that utilized the power of the zebrafish model.

## 2. Comparison of Zebrafish and the Human Heart

Heart development is regulated by molecular, cellular, and environmental factors [10,17,18]. Anatomically, the zebrafish heart is different from the human heart [10,19]. The human heart consists of four chambers separated by a septum and valves, whereas the zebrafish heart contains a single atrium and a single ventricle separated by atrioventricular valves. Despite these anatomical differences, the zebrafish has emerged as a valuable vertebrate model system for cardiovascular study [10,19]. Cellular and molecular mechanisms of heart development are highly conserved between zebrafish and human. Early cardiac morphological events between these two species, including cardiac progenitor formation, assembly of the myocardial plate, heart tube formation, cardiac looping and valve formation are highly similar although the developmental time needed to complete these events in zebrafish is significantly less than in human. In zebrafish, cardiac progenitor formation starts around 5 h post-fertilization (hpf) followed by other events leading to the initiation of valve formation, which starts around 48 hpf. In humans, on the other hand, cardiac progenitors form around 15 to 16 days into embryonic development, and the cardiac cushion, the precursor of valve and septa, form around day 28. The cellular framework of the zebrafish heart is comparable to the mammalian heart. The mammalian heart is developed from contributions of the first heart field (FHF), the second heart field (SHF), and the cardiac neural crest cells [20,21]. Recent studies have shown that zebrafish heart is also formed from contributions of FHF, SHF, and cardiac neural crest cells [10,22,23,24]. Many genes and regulatory networks essential for cardiogenesis in zebrafish are also essential for mammalian cardiogenesis. Zebrafish forward and reverse genetic studies have identified roles of many previously unknown genes in vertebrate cardiac development and function because, unlike mouse and chicken, zebrafish embryos can survive the first 7 days of their development without a functional cardiovascular system, receiving oxygen by passive diffusion through the skin [25]. Moreover, cardiovascular physiology of the human more closely resembles the zebrafish than the rodent [26]. Many electrical properties of the zebrafish heart are similar to the electrical properties of the human heart [10,27,28,29,30]. The zebrafish embryonic heart rate (140–180 beats per minute; bpm) is much closer to the normal fetal heart rate (130–170 bpm), which is very different from mouse heart rate (300–600 bpm) [31]. In recent years, the zebrafish has gained popularity for cardiovascular research because of its capacity to regenerate the heart [32], which provides exciting opportunities to discover new therapies for cardiac injury.

## 3. Advantages of Zebrafish in Cardiotoxicity Study

As a toxicology model, the zebrafish is a promising intact multicellular organism that offers many advantages over traditional cell culture model systems, which are helpful in determining cytotoxicity but fail to recapitulate complex interactions present in the whole organism [13,14,15,16]. The zebrafish represents the true complexity of an intact organism, providing opportunity to access absorption, excretion, and toxicity of chemicals exposed in high-throughput testing. Simple heart-rate measurement of zebrafish can provide predictive information regarding the interaction of chemicals with the components of the cardiac functional regulatory network. Researchers have shown that drugs that produce cardiac toxicity in human by inducing repolarization abnormalities, consistently produced bradycardia in zebrafish, underscoring the similarity between both systems and the utility of the zebrafish in predicting cardiac toxic chemicals [33].

**Figure 1:**
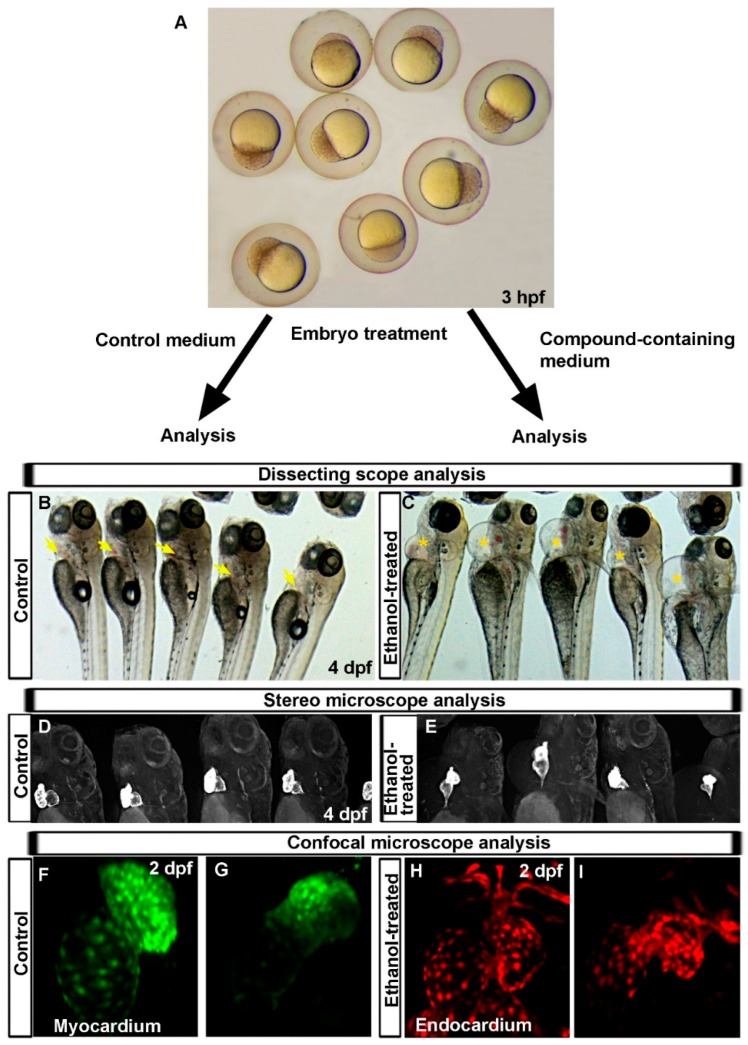
Advantages of use of zebrafish in cardiotoxicity research, which provide enormous information within a short time. (**A**) Dissecting microscope image of 3 hpf zebrafish embryos showing how easily accessible zebrafish embryos are to treat with chemicals at different developmental stages for desired periods; (**B**,**C**) Dissecting microscope images showing normal pericardium in the control embryos (**yellow arrow**) (**B**) and pericardial edema phenotype in 4 days post-fertilization (dpf) ethanol-treated zebrafish embryos (**yellow** **star**) to help predict defective cardiogenesis (**C**); (**D**,**E**) Bright field images of *Tg*(*myl7:GFP*) embryos showing normal shaped two-chambered heart in control (**D**) and an almost linear heart in ethanol-exposed embryos (**E**), confirming heart malformation after ethanol exposure; (**F**,**G**) Confocal images of *Tg*(*myl7:nlsKiKGR*) embryos showing nuclei of cardiomyocytes in closely apposed bean-shaped atrium and ventricle in control embryos (**F**), fewer cardiomyocytes are seen in misshapen chambers of ethanol-treated embryos (**G**); and (**H**,**I**) Confocal images of *Tg*(*fli1:EGFP*) embryos show endocardial cells in normal endocardium in control embryos (**H**), fewer endocardial cells are seen in misshapen endocardium of ethanol-treated embryo (**I**).

The zebrafish is immensely useful to examine chemical toxicity during prenatal development. Externally developing embryos can be easily exposed to chemical by incubating embryos in the desired chemical solution. Developmental timing of chemical exposure can be tightly controlled to examine stage-specific exposure effects; a simple dissecting microscope can provide information on cardiac edema; transparent embryos with transgene expression in the heart that allows for the use of stereomicroscope to quickly evaluate heart formation defects; and advanced imaging facilitates detail analysis at the cellular level (Figure 1A–I). Studies have recapitulated cardiac defects in zebrafish similar to those seen in human patients due to prenatal exposure of various teratogens [34,35,36]. High fecundity, rapid development outside the mother’s body, and well-characterized cardiogenesis stages combined with transparency enabling non-invasive, whole animal imaging make the zebrafish embryo an ideal model system for cardiac teratogenicity screens. Zebrafish embryos can survive several days without active circulation [15], which provides enough time to understand the defects and to dissect cellular and molecular mechanisms.

The cost of zebrafish husbandry is significantly lower than the cost of other vertebrate model systems, like mouse or chicken. Thousands of animals housed in a small space are easy to handle and require minimal maintenance. High-throughput studies can be easily performed that provide reliable statistical evaluations, using a large number of embryos or adult fish at relatively low cost. Rapid development and short maturation period also allow for cost-effective evaluation of embryonic substance exposure at different stages of life until old age.

Almost all tools available to study the cardiovascular system in other model systems are also available to study the zebrafish model. In recent decades, an ample number of transgenic zebrafish lines have been created that express fluorescence proteins labeling myocardium, endocardium, different cardiac lineages, blood, and the vascular system [22,23,37,38,39,40,41,42,43,44] (Table 1). There are also transgenic lines reporting different signaling pathways in cardiogenesis and providing over-expression or blockage of cardiac regulatory genes [17,18]. Together, transgenic zebrafish facilitate precise understanding of the effects of substances on cardiogenesis and cardiac functions. Published literature furnishes necessary knowledge on zebrafish cardiogenesis that expedites cardiotoxicity research. Transparency of the externally developing embryos and availability of advanced high-resolution, time-lapse imaging technology facilitate recording and analysis of live events [45] that help detect anatomical and physiological abnormalities in real time resulting from toxic substance exposure. For high-throughput assays, tools are available that handle loading of larvae from a bulk reservoir, positioning, orientating for optimal imaging of desired tissue or organ, image capturing, and finally ejecting the embryos and repeating the process [46]. Advanced electrocardiogram recording tools and optimized protocols are available to detect alteration of cardiac function [47]. Although the microarray is not a simple assay for a small fish embryo or for its even smaller heart, researchers have utilized this technology and identified genes involved in cardiac development and in mediating cardiotoxicity in the embryonic heart [48,49,50,51]. Gene expression profiling using a small zebrafish sample has become possible due to recent advances in technology such as next-generation sequencing and RNA-seq [52,53,54]. Proteomics is also used in zebrafish toxicology research [55].

## 4. Toxic Substances Causing Heart Defects in Zebrafish

Because of these advantages, the zebrafish embryo has emerged as a unique model system to study the effects of various compounds on cardiovascular development and function. Adult zebrafish also provide opportunities to understand acute and chronic compound exposure effects on cardiac function.

### 4.1. Substances Causing Cardiac Development Defects

The zebrafish embryo has been used to assay the impact of exposure to nanoparticles during development [56]. A zebrafish study comparing the toxicity of gold, silver, and platinum nanoparticles during development revealed accumulation of metals inside the developing embryo that caused a serious threat to the organism. Silver-nanoparticle exposure produced abnormal cardiac morphology, pericardial edema, and circulation defects in addition to other developmental defects [57].

The toxic effect of the organic pollutant, dioxin, on human health is well known [58]. The effect of one of the most commonly occurring dioxins, 2,3,7,8-tetrachlorodibenzo-*p*-dioxin (TCDD), was investigated in zebrafish [59,60,61]. In addition to other developmental defects, zebrafish embryos exposed to TCDD displayed malformed heart, defective atrioventricular valves, and pericardial edema [62]. It has been proposed that dioxin-like chemicals including TCDD induce toxicity by binding to the aryl hydrocarbon receptor (AhR) [62].

Another class of ubiquitous organic compounds is halogenated carbazoles. Dong and his group [6] tested cardiotoxicity of six carbazole compounds and found that out of the six carbazoles, two, namely 2,7-dibromocarbazole and 2,3,6,7-tetrachlorocarbazole, were more toxic than the rest. These two carbazoles produced phenotypes similar to dioxin-induced phenotypes, including pericardial edema and straight elongated hearts in zebrafish embryos at nanomolar concentrations. Morpholino knockdown of the aryl hydrocarbon receptor 2 (AhR-2) gene rescued carbazole-induced defects, which indicates that the acute cardiotoxicity was AhR dependent [6].

Firemaster 550 (FM550), a primary fire retardant, is a mixture of brominated and aryl phosphate ester components. FM550 ingredients are not only found in the outdoor environment, but ubiquitously detected at much higher concentration in an indoor environment [63]. McGee and his colleagues used zebrafish to screen developmental toxicity of each of the components of FM550 and showed that exposure to brominated components produced no significant effect on embryonic development [64]. However, two aryl phosphate ester (APE) components, triphenyl phosphate (TPP) or mono-substituted isopropylated triaryl phosphate (mono-ITP) that comprise 50% of FM550 resulted in cardiac defects producing a tube-like heart [64]. Investigation of sensitive developmental windows for APE-induced cardiotoxicity identified early embryonic stages until pharyngula or the phylotypic stage (24 hpf), a developmental period prior to completion of cardiac looping, are more susceptible to the cardiac looping defect. Since the APE-treated embryos resemble embryos treated with the AhR agonists, McGee et al. [64] hypothesized that APE-induced heart defects were AhR mediated. To investigate the mechanisms, the authors blocked AhR by either co-treating embryos with the AhR antagonist (CH223191) or by knocking down AhR2 by injecting AhR2-specific morpholino and treating the embryos with APE. Blocking of AhR by CH223191, not by AhR2 knockdown, blocked cardiac looping defects following exposure to mono-ITP. TPP-induced defects were not rescued by AhR blocking, suggesting both APEs mediate cardiac defects through different pathways [64]. Later, Gerlach and his colleagues knocked down all three known zebrafish AhR isoforms and showed mono-ITP-mediated cardiotoxicity was the result of an AhR-independent pathway, which was also antagonized by CH223191 [65].

The polycyclic aromatic hydrocarbon, phenanthrene, is the most abundant in the environment and is a known cardiotoxic agent. The zebrafish was used to investigate the mechanisms of phenanthrene-induced cardiotoxicity. Environmentally relevant concentrations of phenanthrene treatment for 72 h produced pericardial edema, abnormal heart looping and an enlarged ventricle with a thinner ventricular wall. Zhang et al. showed that phenanthrene treatment significantly increased mRNA and protein expression levels of matrix metalloproteinase-9 and its activity. Treatment of metalloproteinase-9 inhibitor attenuated phenanthrene-induced cardiac defects [66]. Cytochrome P450 1A (Cyp1a) inhibition by morpholino oligonucleotide combined with zebrafish exposure to weak aromatic hydrocarbon receptor agonist phenanthrene showed cardiac toxicity, but these combined effects are not rescued by AhR knockdown [67].

Complex mixtures of environmental pollutants are often encountered in nature. Zebrafish was used to model exposure of petroleum-derived product pollution from stormwater runoff, aquatic sediments in urbanized areas or oil spills in aquatic environments using zebrafish embryos, showing differential roles of AhRs for various aromatic hydrocarbons in petroleum-derived product toxicity [68,69]. In addition, zebrafish was used to show that bioretention filtration is effective at preventing toxicity of petroleum-derived products [70,71].

Chronic exposure to paclobutrazol, a triazole-containing fungicide widely used in agriculture, was reported to affect reproductive, antioxidant defense, and liver metabolism systems in animals [72,73,74,75,76]. Its effects during development were examined in the zebrafish. Wang and colleagues showed that paclobutrazol disrupted heart development by affecting cardiac looping and produced pericardia edema [77].

**Figure 2:**
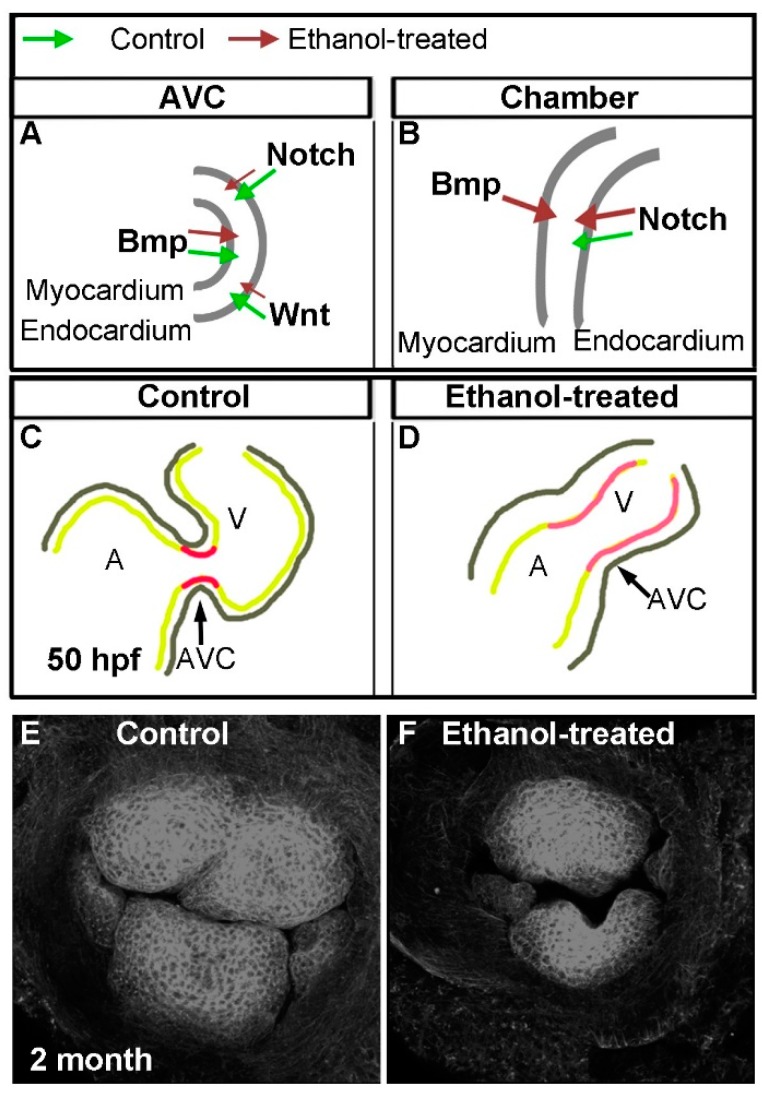
Zebrafish studies discovered altered valve regulatory pathways due to embryonic ethanol exposure leading to persistent atrioventricular valve defects. (**A**) Schematic representation of atrio-ventricular canal (AVC) showing myocardium and endocardium layers. Bmp, Notch and Wnt signaling play critical roles during AVC differentiation. Ethanol exposure reduced Notch and Wnt activity at the AVC (represented by small **dark brown arrows**) during atrioventricular valve formation; (**B**) Schematic representation of the ventricle showing myocardium and endocardium layers. Ethanol exposure (3–24 hpf) increased Notch activity in the ventricle (represented by big dark brown arrows) during atrioventricular valve formation. **Green arrow**: normal condition; **dark brown arrow**: ethanol-exposed condition; (**C**) Schematic representation of atrium, ventricle and AVC (**black arrow**) of the control zebrafish heart at 50 hpf (during atrioventricular valve formation). Differentiated valve-forming cells (**red**) are localized at the AVC. Gray line represents myocardium layer; greenish-yellow line represents endocardial layer; (**D**) Schematic representation of the atrium, the ventricle and the AVC of the ethanol-treated (3–24 hpf) zebrafish heart at 50 hpf (during atrioventricular valve formation). Note that the shape of the heart is different from control. Differentiated valve-forming cells (**pinkish-red**), which do not exhibit all characteristics of normal valve cells are not restricted at the AVC. Those cells extend into the ventricle. The distance between myocardium and endocardium (the space containing cardiac jelly; **black line**) is more in ethanol-treated embryos. Gray line represents myocardium layer; greenish-yellow line represents endocardial layer; and (**E**,**F**) Wheat germ agglutinin-stained atrioventricular valves of two-month-old zebrafish shows four well-organized valve cusps in control fish (**E**), and small, deformed valve cusps in fish treated with ethanol during embryonic development (3–24 hpf).

Prenatal alcohol exposure leads to a range of birth defects including various congenital heart defects (CHDs). Mechanisms of alcohol exposure-associated CHDs are not understood. It is also not known whether alcohol interferes with a single critical event or with multiple events during cardiac development. Zebrafish embryos are an ideal model to ask these questions because of the external development of the embryos, well-characterized heart developmental stages, and easy treatment of the embryos with ethanol at different heart development stages. Continuous ethanol exposure from a single cell until the hatching period (2–3 dpf) altered morphology and function of the zebrafish heart [78]. Using various transgenic lines to label myocardial and endocardial cells and advanced microscopy techniques, Sarmah and Marrs showed that ethanol exposure perturbed all cardiogenic stages tested, including cardiac specification, myocardial migration, looping, chamber morphogenesis, and endocardial cushion formation [35]. Short-term exposures (either from gastrulation until cardiac specification or during myocardial midline migration) did not produce persistent heart development defects in zebrafish within the limitations of the experiments. However, longer-exposure at different developmental stages produced aberrant heart looping, defective chambers as well as defective endocardial cushions, which are precursors of heart valves and septa (in a more than two-chambered heart). The severity of heart defects varies with the stage, duration, and concentration of ethanol exposure. Analyses of cardiac specification regulatory network revealed temporal and spatial mis-expression of genes including *hand2*, *gata5*, *fgf8a*, *myl7*, and *vmhc* in ethanol-exposed embryos [35]. Interestingly, this study showed that endocardial cushion formation was specifically sensitive to embryonic ethanol exposure. Ethanol withdrawal long before endocardial cushion formation led to valve defects. Following this study, Sarmah and colleagues combined state-of-the-art zebrafish transgenic lines that label myocardium and endocardium with various cardiac regulatory signaling reporter lines including Bmp, Notch, and Wnt signaling reporters, marker staining, and advanced microscopy to gain mechanistic insight into the etiology of ethanol-induced atrioventricular valve defects [36]. This study showed that ethanol exposure reduced Bmp signaling during early heart developmental stages. But later during endocardial cushion formation, when control embryos suppressed Bmp activity from the chamber cardiomyocytes and intensified the activity at the atrioventricular canal (AVC), ethanol-exposed embryos had Bmp activity throughout the ventricle (Figure 2A,B). Similarly, ethanol exposure caused redistribution of Notch active cells in the heart, leading to reduced Notch activity at the AVC but ectopic activation of Notch signaling in the ventricle chamber (Figure 2A,B). Ethanol exposure reduced Wnt activity during endocardial cushion differentiation (Figure 2A). Those ethanol-exposed embryos showed aberrant valve formation during development and defective valves at the juvenile stage (Figure 2C–F). Valve leaflets were smaller, irregularly shaped, and did not closely correspond with each other to allow complete closure of the atrioventricular junction (Figure 2E,F) [36].

Smoking cigarettes and the use of tobacco products continue to be serious problems worldwide, causing severe illnesses from cancer to heart diseases and premature deaths. Like maternal drinking, maternal tobacco smoking has been consistently associated with increased risk of CHDs in the fetus [79,80,81]. Tobacco smoke is a mixture of more than 5000 toxic and carcinogenic chemicals [82,83]. Nicotine, a potent stimulant present in the tobacco plant, is the addictive substance in tobacco. Zebrafish embryos were used to assess the effects of tobacco smoke and e-cigarette aerosol extracts on vertebrate cardiac development and function [84]. Zebrafish embryos were treated with nicotine, cigarette or e-cigarette extracts at concentrations 6.8, 13.7, and 34 μM nicotine from cleavage stage until 3 dpf, and heart development was examined at 3 dpf. The study showed that the cigarette and e-cigarette extracts produced malformed hearts, showing phenotypes that include looped heart with slight pericardial edema, un-looped heart with pericardial edema, and stretched un-looped heart with no directional blood flow. Their data indicated that both e-cigarette and tobacco cigarette smoke extract exposure affected heart development with more severe defects for the exposure to tobacco cigarette smoke, but similar concentrations of nicotine exposure alone did not produce significant heart development defect [84]. However, higher concentrations of nicotine exposure (1.3–1.7 mM) during embryonic development (exposure periods 4–24 hpf or 4–48 hpf) led to heart developmental defects [34]. Pericardial edema and various degrees of heart defects, from near-normal morphology to severe pericardial edema, were seen at 3 dpf after nicotine exposure to zebrafish embryos. Severity of the defects increased as the concentration of nicotine increased. Statistical analysis showed a strong positive correlation between nicotine dose and heart defect severity [34].

### 4.2. Substances Causing Cardiac Function Defects in Larvae

The zebrafish embryo and larvae have been used to study chronic as well as acute substance exposure effects on heart function. A zebrafish study has shed light into how the low-doses of silica nanoparticles (SiNPs) exposure affect cardiac function [48]. The average diameter of SiNPs tested was approximately 62.14  ±  7.16 nm. The investigators injected nanoparticles into the duct of Cuvier of 48 hpf embryos and analyzed SiNPs effect on heart function at 72 hpf. Injection of different concentrations of SiNPs caused bradycardia and reduced cardiac output without causing any blockage in the atrioventricular canal. Microarray analyses revealed that SiNP exposure induced neutrophil-mediated inflammation and reduced cardiac contraction by reducing the expression of cardiac contraction protein TNNT2 and by inhibiting the calcium signaling pathway [48].

Widely used organophosphate and carbamate pesticides, which are acetylcholinesterase inhibitors, can be toxic to vertebrates because of the conservation of acetylcholinesterase between invertebrates and vertebrates. Experiments examined three commonly used organophosphate pesticide—chlorpyrifos, dichlorvos, and diazinon—by exposing zebrafish embryos in a range of pesticide concentrations up to 1 mM. Chlorpyrifos and dichlorvos exposure reduced heart rate in a dose-dependent manner. Diazinon did not change the cardiac motor activity in zebrafish but produced cardiac edema [85].

The effect of carbamate pesticide Sevin™, a commonly used insecticide containing carbaryl (1-napthyl-*N*-methylcarbamate) as an active ingredient, was analyzed during zebrafish heart development. Although chronic carbaryl exposure did not lead to malformed heart, the exposure induced bradycardia in zebrafish embryos [86]. Similar to chronic exposure, acute exposure of 100 µg/mL carbaryl for 10 min reduced the heart rate of embryos. Other studies, including a more recent study, showed heart malformation effects of zebrafish carbaryl exposure [87]. The fungicide paclobutrazol exposure during development also reduced heart rate, but the reduction may be associated to the paclobutrazol-induced defective heart structure [77].

Organic pollutant dioxin exposure not only causes heart malformation, it also leads to defects in cardiac function. Dioxin compound TCDD-treated embryos had slow heart rate, reduced cardiac output, and regurgitation of blood flow at the atrioventricular valve [62]. The fire-retardant FM550 components, APEs, when exposed to embryos after cardiac looping did not result in significant heart malformations in contrast to the embryos exposed to APE prior to cardiac looping, but those embryos had significantly reduced (~50%) heart rate. The magnitude of heart rate reduction was comparable to the reduction seen in the embryos having APE-induced cardiac looping defect [53]. Aromatic hydrocarbon (phenanthrene) exposure during embryonic development also affected cardiac function of zebrafish larvae and increased heart rate dramatically [66].

The acute effect of cocaine, a recreational drug, on heart rate and blood pressure has been studied in many animal models, including human. It is known that lower doses of cocaine cause tachycardia but higher doses lead to bradycardia in humans and in other animal species [88]. Darland and his [89] group investigated acute effects of cocaine on the heart rate of zebrafish larvae. Five-day-old larvae exposed to cocaine at different concentrations displayed a bell-shaped dose response curve, tachycardia at lower doses and bradycardia at higher doses, showing similar response to cocaine as human [89].

Zebrafish embryos were modeled to study the toxicity of chronic cigarette smoke exposure by treating embryos with cigarette smoke condensate of different commercial brand cigarettes (two reference and six Canadian brand cigarettes with different design features). Exposure to the cigarette smoke condensates of all brands from 24–48 hpf with heartbeat measurement at 48 hpf showed reduction of embryo’s heartbeat by 50% [2]. Jensen and colleagues compared the toxicity of cigarette smoke and snuff extracts by continuously exposing 48 hpf embryos to either cigarette smoke extract or snuff extract for 24 h. Assessment of cardiac functions at 72 hpf revealed that exposure to both mixtures led to the reduction of both systolic and diastolic volumes, decrease in heart rate, stroke volume, and cardiac output [90].

There was a belief that the harmful effects of cigarette smoke come mainly from the mixture of toxic and carcinogenic chemicals present in the tobacco smoke [2,84]. Since the cause of tobacco addiction is nicotine, nicotine patches have been offered to smokers to help them quit smoking. Acute effects of nicotine on the function of heart were examined using 3 dpf zebrafish embryos [34]. Embryos were exposed to different concentrations of nicotine solutions (50 µM, 100 µM, and 1.2 mM) and heart rates were measured after 5 min of nicotine exposure. Like cocaine, nicotine exposure showed dose-dependent effect on heart function, causing tachycardia at lower doses and bradycardia at higher doses [34], resembling the types of arrhythmia previously observed in other model organisms [91]. However, chronic exposure to nicotine from 2–48 hpf reduced the heartbeat of embryos at later stages, an effect possibly due to the malformed heart [34].

Zebrafish embryos were also used to study prenatal ethanol exposure effect on cardiac function. Chronic alcohol exposure until heart tube formation not only led to a malformed heart but also led to minor tachycardia, suggesting altered heart function due to prenatal alcohol exposure [36].

### 4.3. Cardiotoxicity Due to Exposure to Adult

There are a great deal fewer toxicological studies using juvenile and adult zebrafish than zebrafish embryos, highlighting an opportunity for additional research and toxicology model development. Cocaine consumption leads to a dose-dependent change of heart rates in humans. Heartbeat is increased if a low dose is consumed, but it decreases if a high dose is consumed. The effect of cocaine on the physiology of zebrafish was studied by electrocardiograms (ECGs). Adult zebrafish treated with various cocaine doses followed by ECG showed similar patterns in ECG data as seen in various mammals: a bell-shaped dose response curve showing initial increase in heart rate with lower doses followed by reduced heart rate at higher doses [89].

An acute exposure to the polycyclic aromatic hydrocarbon benzo-*a*-pyrene, by intraperitoneal injection or aquatic exposure in adult zebrafish caused cardio-respiratory impairment, leading to increased oxygen demand and reduced ventricular heart rate [92].

## 5. Conclusions

Because of the conservation between zebrafish and human cardiogenesis, as well as the other advantages of zebrafish mentioned above, the zebrafish model has already become an invaluable system to study human cardiovascular development and disease. Use of the zebrafish as a toxicology model to study cardiotoxicity has only begun. We have presented here the use of zebrafish in testing various compounds, ranging from organic and inorganic pollutants to drugs of abuse. Within a relatively short time, the effects of novel compounds that are widespread in the environment on cardiogenesis were assessed using zebrafish. Zebrafish studies shed light on illicit drug exposure-associated cardiovascular developmental defects (CHDs). CHDs seen in human patients because of prenatal alcohol exposure as well as the cardiac function defects due to cocaine consumption or smoking were successfully modeled in zebrafish. New studies have started providing insights into the molecular mechanisms of the defects associated with illicit drug exposure. Zebrafish studies also have identified toxic effects of pesticides, nanoparticles, and organic pollutants on cardiac development and function. Cardiac toxicity due to 2,3,7,8-tetrachlorodibenzo-*p*-dioxin (TCDD) or halogenated carbazole exposure was linked to the binding ability of these ligands with the aryl hydrocarbon receptor (AhR). However, studies done to test most other chemicals were exploratory. Many studies used pericardial edema and heart rate as endpoint analyses for cardiac toxicity. Pericardial edema provides general information on cardiotoxicity and abnormal heart rate indicate defective cardiac function. However, these measures do not provide definitive mechanistic information. The zebrafish model offers an outstanding opportunity to execute detailed cellular and molecular analyses to dissect cardiac development mechanisms sensitive to toxic chemical exposure or to analyze dysregulated pathways leading to defective cardiac function. The full potential of zebrafish should be exploited in future studies to decipher molecular mechanisms disrupted due to specific chemical exposure, which will generate more mechanistic understanding and help to design studies that remedy the defects.

Until now, zebrafish larvae have been explored largely in toxicity research, including cardiotoxicity. Zebrafish larva has certainly been a strength of this model organism, but adult zebrafish has also been recently used, which shows the potential of this model organism in adult cardiotoxicity research. More research is needed to better understand the anatomy and physiology of the adult zebrafish heart to allow it to emerge as a better toxicology model. It is worthwhile to point out that the zebrafish will not replace the role of other mammals in preclinical toxicology research. The exposure method to zebrafish, which is mainly bathing, is very different from the exposure method to human or other mammals. Consequently, absorption, half-life and secretion of the compound in zebrafish will be different from mammals. Moreover, zebrafish detoxifying and drug-metabolizing enzymes are not fully characterized yet. However, like cell culture systems, zebrafish can be exploited as an early stage model in preclinical drug studies to eliminate toxic compounds, which will help prioritize lead molecules.

## Figures and Tables

**Table 1:** Transgenic zebrafish lines that mark different cell types of the heart.

Transgene Name	Cell Label	Description of the Expression	Reference
*Tg*(*myl7:GFP*)	Cardiomyocytes	GFP in the cytoplasm of differentiated cardiomyocytes.	[39]
*Tg*(*myl7:nucDsred*)	Cardiomyocytes	DsRed in the nuclei of differentiated cardiomyocytes.	[43]
*Tg*(*myl7:ras-eGFP*)	Cardiomyocytes	Enhanced GFP in the cell membrane of differentiated cardiomyocytes.	[38]
*Tg*(*myl7:nlsKikGR*)	Cardiomyocytes	KikGR in the nuclei of differentiated cardiomyocytes. UV light exposure photoconverts KikGR from green to a red fluorophore.	[23]
*Tg*(*fli1:EGFP*)	Endothelium and endocardium	Enhanced cytoplasmic GFP in the entire vasculature and in the endocardial cells.	[40]
*Tg*(*fli1:nEGFP*)	Endothelium and endocardium	Enhanced nuclear GFP in the entire vasculature and in the endocardial cells.	[42]
*Tg*(*kdrl:GFP*)	Endothelium and endocardial	GFP in the entire vasculature and in endocardial cells.	[37]
*Tg*(*kdrl:nlsKikGR*)	Endothelium and endocardial	KikGR in the nuclei of endothelia and endocardial cells. UV light exposure photoconverts KikGR from green to a red fluorophore.	[23]
*Tg*(*gata1a:DsRed*)	Red blood cells	*DsRed* in red blood cells	[44]
*Tg*(*nkx2.5:nZsYellow*)	*nkx2.5* positive cells	*ZsYellow* in *nkx2.5* positive cells allows lineage tracing of second heart field progenitors.	[41]
*Tg*(*NC:mCherry*)	Cardiac neural crest cells	A double transgenic for the *sox10:GAL4-UAS-Cre* and the *ubi:Switch* reporter. *sox10* promoter drives the expression of Cre recombinase in neural crest cells which excises GFP and permanently labels cells of *sox10* lineage with mCherry and allows lineage tracing of cardiac neural crest cells.	[22]
*Tg*(*NC:NfsB-mCherry*)	Cardiac neural crest cells	A double transgenic for *sox10:GAL4-UAS-Cre* and *UAS:NfsB-mCherry* in which the expression of Nitroreductase-mCherry fusion protein is controlled by *sox10*-driven GAL4 activity allowing lineage tracing of cardiac neural crest cells.	[22]
